# Effects of a Novel Poly (AA-*co-*AAm)/AlZnFe_2_O_4_/potassium Humate Superabsorbent Hydrogel Nanocomposite on Water Retention of Sandy Loam Soil and Wheat Seedling Growth 

**DOI:** 10.3390/molecules171112587

**Published:** 2012-10-25

**Authors:** Shaukat Ali Shahid, Ansar Ahmad Qidwai, Farooq Anwar, Inam Ullah, Umer Rashid

**Affiliations:** 1 Department of Physics, University of Agriculture, Faisalabad 38040, Pakistan; 2 Department of Physics, University of Karachi, Karachi 75270, Pakistan; 3 Department of Chemistry, University of Sargodha, Sargodha 40100, Pakistan; 4 Department of Chemistry and Biochemistry, University of Agriculture Faisalabad-38040, Pakistan; 5 Institute of Advanced Technology, Universiti Putra Malaysia, Serdang-43400, Selangor, Malaysia

**Keywords:** poly (AA-*co*-AAm)AlZnFe2O4/K-H, superabsorbent hydrogel nanocomposite characterization, SEM, EDX, FTIR, soil amendment, seedling growth

## Abstract

A novel poly(acrylic acid-*co*-acrylamide)AlZnFe_2_O_4_/potassium humate superabsorbent hydrogel nanocomposite (PHNC) was synthesized and its physical properties characterized using SEM, Energy Dispersive X-ray (EDX) and FTIR spectroscopic techniques. Air dried sandy loam soil was amended with 0.1 to 0.4 w/w% of PHNC to evaluate its soil moisture retention attributes. Effect of PHNC amendment on pH, electrical conductivity (EC), porosity, bulk density and hydraulic conductivity of sandy loam soil was also studied. The soil amendment with 0.1 to 0.4 w/w% of PHNC remarkably enhanced the moisture retention at field capacity as compared to the un-amended soils. Seed germination and seedling growth of wheat (*Triticum aestivum* L.) was considerably increased and a delay by 6–9 days in wilting of seedlings was observed in the soil amended with PHNC, resulting in improved wheat plant establishment and growth.

## 1. Introduction

Water conservation is a key step to attaining sustainable agriculture growth and development and productivity. The use of hydrophilic polymers, commonly known as superabsorbents (SAPs), to improving soil water and fertilizer retention properties and thus crop productivity is attracting considerable interest [1,2,3,4,5]. Different clays such as kaolin, bentonite, montmorillonite, attapulgite, smectite and cellulose nanowhiskers have so far been used in the synthesis of superabsorbent hydrogel composites (SHCs) [6,7,8].

Humic compounds not only improve absorption of microelements but also enhance photosynthesis and root development [9]. Humic substances (HS), produced due to organic matter decomposition, are known to be the natural compounds containing 50 to 90% of the organic matter of peat, lignites, sapropels, and non-living organic matter of soil and water. It is widely accepted that these substances are one of the most potential sources of the humates used in agriculture [9,10].

Research has confirmed that HS can indirectly and directly affect the physiological processes of plant growth. They provide minerals, increase the micro-organism population, provide biochemical substances, and carry trace elements and growth regulators [10]. Application of humic acids (as one of the main fractions of humic substances) in agriculture as soil fertilizer and soil conditioner has been extensively discussed in the literature [10,11]. The HA products are usually available in the form of inexpensive soluble salts, referred to as potassium humates [11]. The K-humate derived from lignite brown coal which is aromatic in nature and contains plenty of carboxylic and phenolic groups, provides favorable conditions for chemical reactions, biological activity and increase pH buffering, improves physical structure of soil and accelerate transport of nutrients to plants [12].

For the development of suitable polymers for soil water conservation, natural clays [13,14] have been used as nanocomposite materials by several researchers to enhance the physical properties of superabsorbent hydrogels [13,14]. However, some crop nutrients such as zinc (Zn) and iron (Fe) that enhance the crop yield and quality [15,16] may be incorporated in the superabsorbent hydrogel to get larger nutrient surface areas enhancing their availability to the plant roots [17,18,19,20,21]. Moreover, potassium humate can be exfoliated to get further improvement in soil physical properties and biological activities and accelerate transport of nutrients to plants [12]. Therefore, it was planned to synthesize a new super absorbent hydrogel nanocomposite material and study the moisture retention characteristics of PHNC amended sandy loam soils. The growth attributes of wheat in soils amended with poly(AAm–co-AA)/AlZnFe_2_O_4_/K-H super absorbent hydrogel nanocomposite (PHNC) were also studied.

## 2. Results and Discussion

### 2.1. Characterization of PHNC

**Figure 1:**
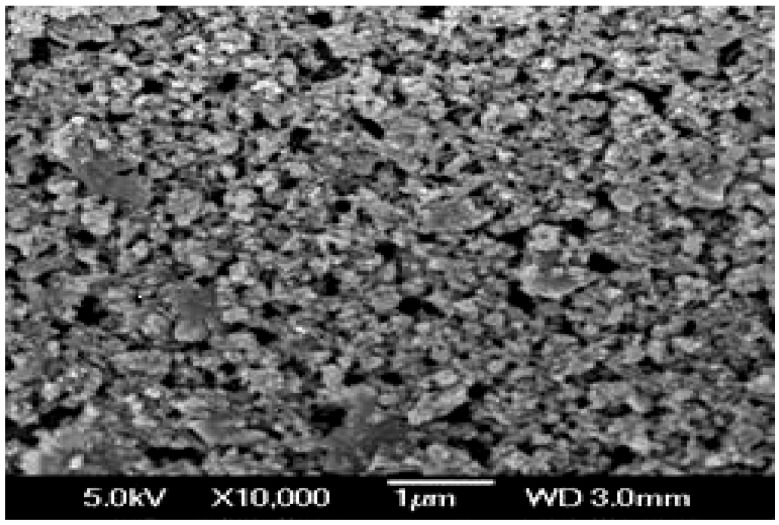
SEM analysis of the AlZnFe_2_O_4_.

**Figure 2:**
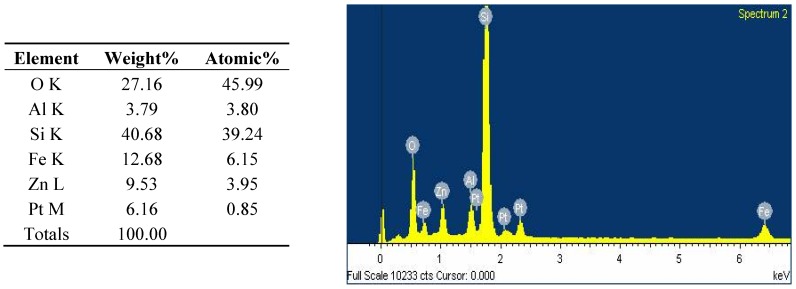
EDX spectrum of AlZnFe_2_O_4_ nanocomposite.

**Figure 3:**
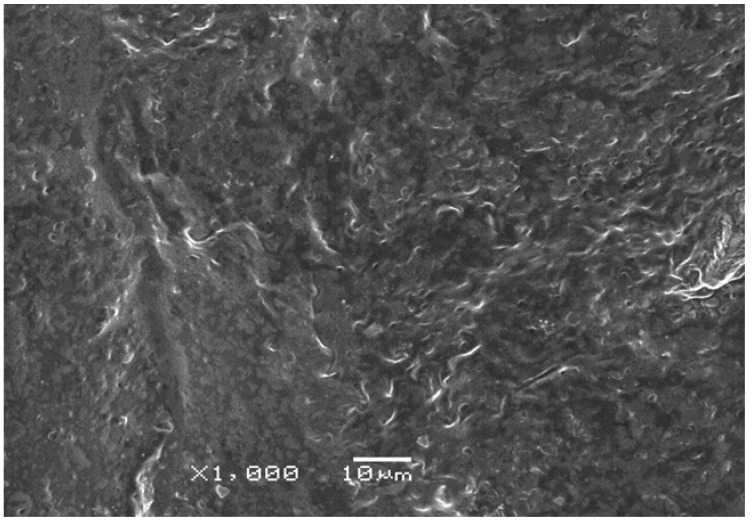
Scanning electron micrograph of PHNC.

A novel poly(acrylic acid-*co*-acrylamide)AlZnFe_2_O_4_/K-H superabsorbent hydrogel nanocomposite was synthesized for agricultural use and SEM analysis of the synthesized nanoparticles was carried out (Figure 1). This analysis showed that the mean diameter of the nanoparticles is 50 nm. The chemical composition of the nanoparticles was determined by Energy Dispersive X-ray analysis (EDX), which showed that nanoparticles contain only Fe, Zn, Al and O with no traces of by-products (Figure 2). A scanning electron micrograph of superabsorbent PHNC is shown in Figure 3.

#### FTIR Spectroscopy

**Figure 4:**
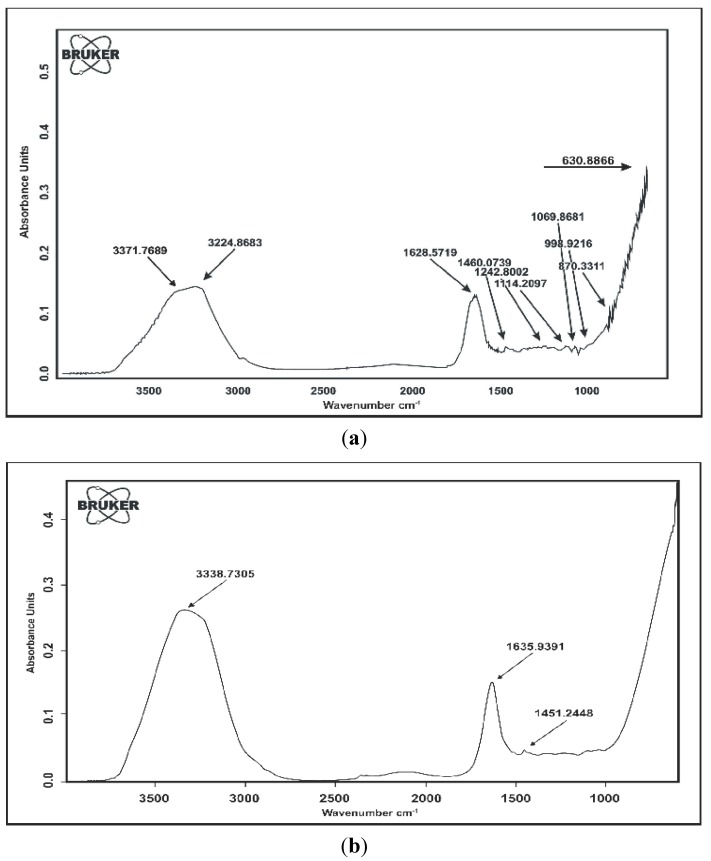
(**a**) FTIR spectrum of poly(acrylic acid-*co*-acrylamide); (**b**) FTIR spectrum of poly(Acrylic Acid-*co*-Acrylamide)/AlZnFe_2_O_4_/K-H.

It is evident from the FTIR spectrum of the superabsorbent hydrogel that two N-H stretching bands appear at 3224.8683 and 3371.7689 cm^−1^, respectively. The C=O stretching is also observed at 1628.5719 cm^−1^. The peak at 1460.0739 cm^−1^ is the C-N stretching band and 1114.2097 cm^−1^ is another peak related to the amide group. The peak appearing at 1242.8002 cm^−1^ is the characteristic (C-O) stretching peak of -COOH (Figure 4a).

When the spectrum of PHNC is compared with that of superabsorbent hydrogel, it can be observed that the N-H stretching bands of the -NH_2_ group are shifted to 3,338.7305 cm^−1^, while the C=O stretching and the C-N are shifted to 1635.9391 and 1451.2448 cm^−1^, respectively, indicating NH_2_ degradation, hydroxyl formation and relatively weaker intensity of peaks due to composite formation (Figure 4b).

### 2.2. Effect of PHNC on Moisture Retention in Soil

**Figure 5:**
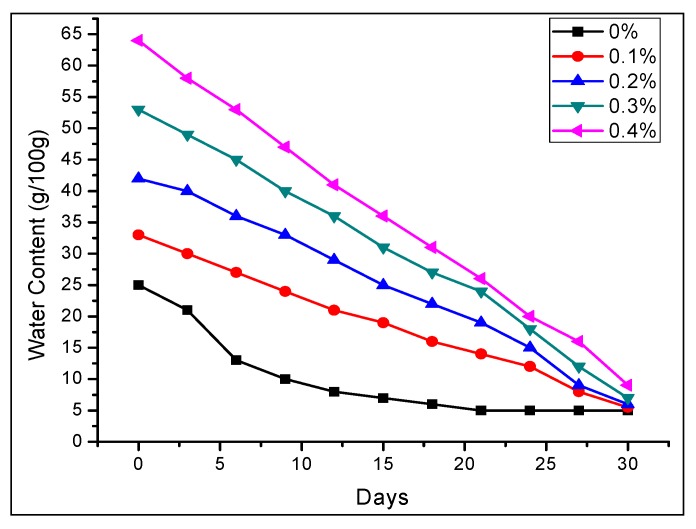
Water retention by the soil amended with different levels (0.1, 0.2, 0.3, 0.4 w/w%) of PHNC levels on water content at field capacity.

It is obvious from Figure 5 that the increase in water retention of soil depends on the quantity of the PHNC used and the highest value of moisture retention was achieved with the addition of 0.4 w/w% PHNC in the soil. In close agreement to our present data, Dorraji *et al*. [22] achieved moisture retention as high as 0.6 w/w% with the use of hydrophilic polymer in sandy and loamy soils.

The sandy loam soil amended with 0.1 to 0.4% of PHNC possessed a good capacity for water retention at field capacity (0.03 MPa pressure) even four weeks after initial watering (Figure 5) [2,6,8]. This remarkable increase in soil water retention might be attributed to the hydrophilic polymer network and introduction of adequate amount of potassium humate in the superabsorbent hydrogel polymeric network which enhanced hydrophilicity of PHNC. However, a gradual decrease in water contents of soil amended with 0.1 to 0.4 w/w% PHNC was observed which finally approached 5 to 8 g/100 g after 30 days. This gradual decrease in water contents can be linked to the water uptake by the plants and some transpiration. The previous studies reported in the literature verify this phenomenon [2,22,23].

### 2.3. Effect of PHNC on Soil pH and Electrical Conductivity (EC)

**Figure 6:**
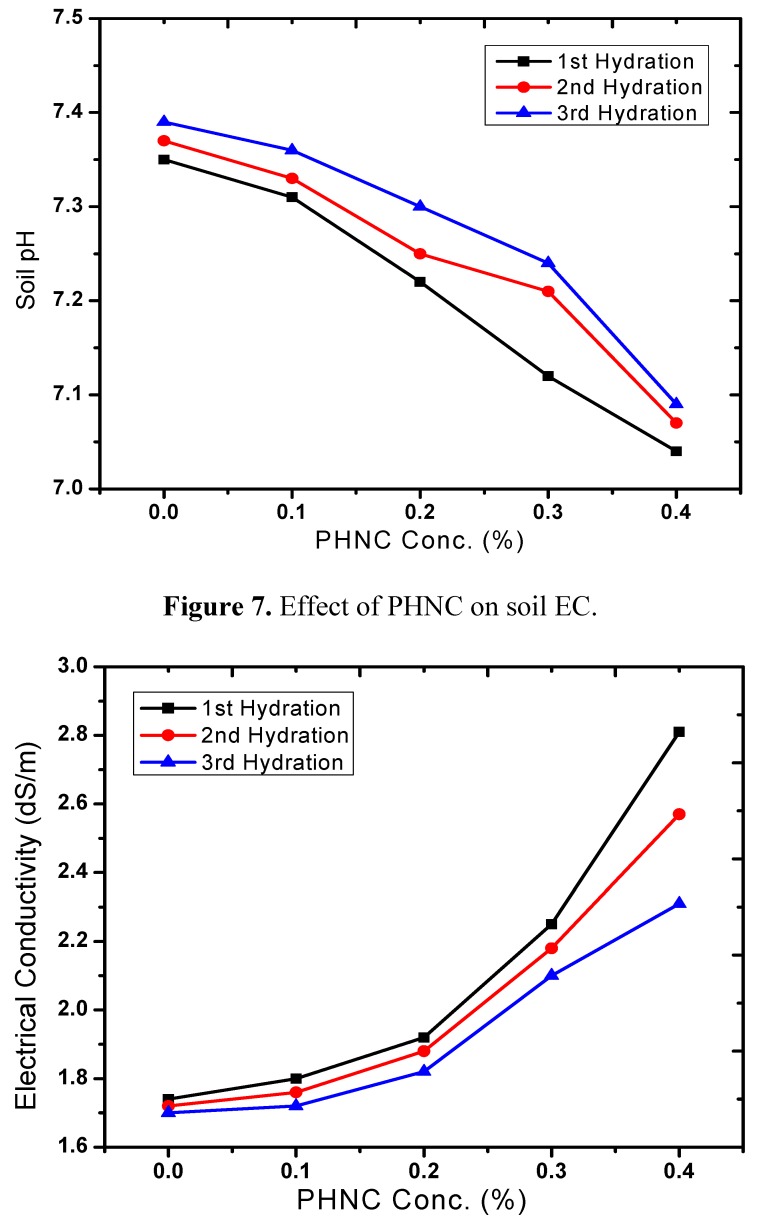
Effect of PHNC on soil pH.

**Figure 7:**
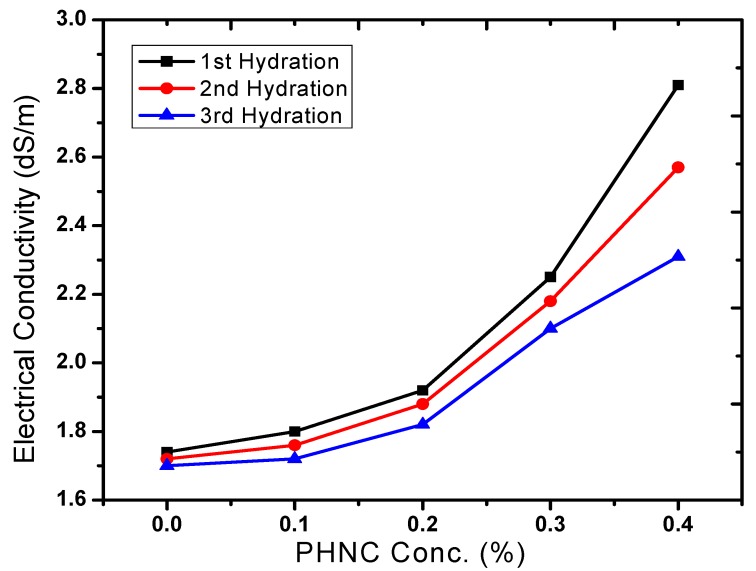
Effect of PHNC on soil EC.

The pH and EC are important factors of the soil chemical, physical and biological properties [23]. The pH and EC varied with the application of PHNC (Figure 6 and Figure 7). Soil pH was reduced by 2 to 5% at concentration of 0.1 to 0.4%, compared with the control. Meanwhile, the electrical conductivity (EC) of PHNC amended soil increased about 6 to 57% (after 1st, 2nd and 3rd hydration) as compared to the control at concentrations 0.1 to 0.4%. The decrease in pH of the soil might have enhanced the discharge of soil inorganic salts thereby increasing EC of the soil. Similar effect on the pH and EC of soils due to the chemical structure of the superabsorbent polymer and soil characteristics has previously been appraised by Liu *et al*. [19] and Bai *et al*. [24], while studying the characteristics of chitosan-graft-poly (acrylic acid)/sodium humate superabsorbent.

### 2.4. Effect of PHNC on Soil Bulk Density and Porosity

**Figure 8:**
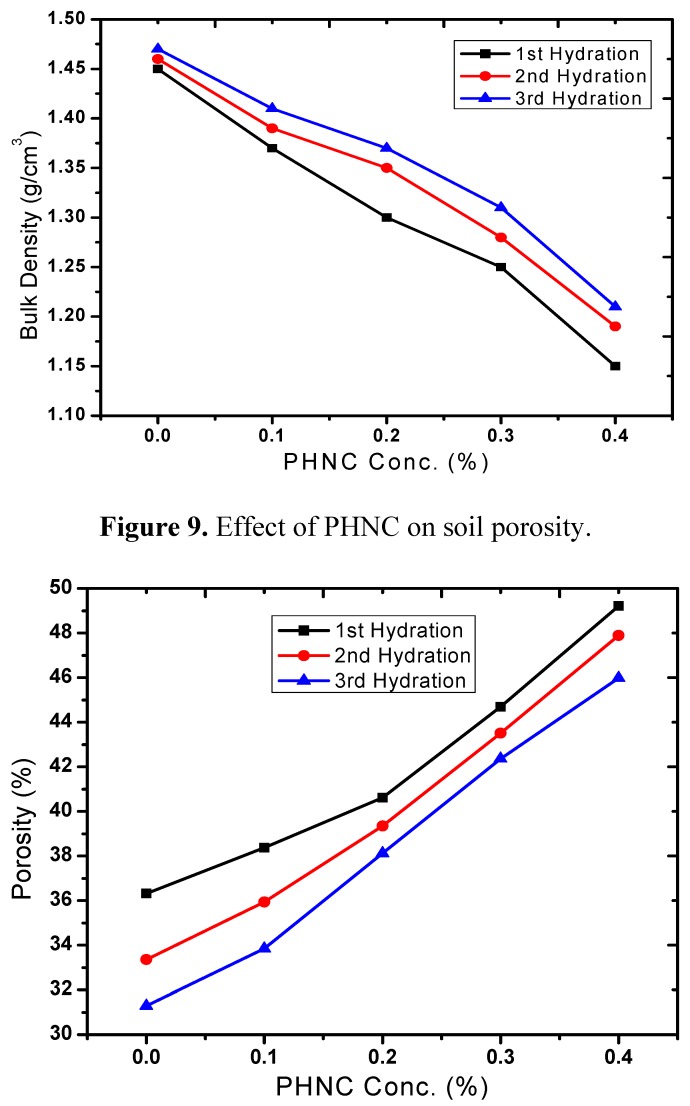
Effect of PHNC on soil bulk density.

**Figure 9:**
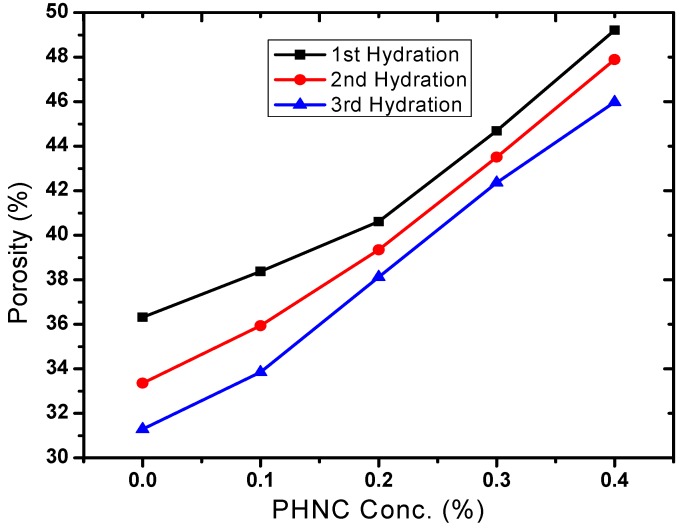
Effect of PHNC on soil porosity.

Bulk density has relationship with other properties of the soil such as porosity, moisture, and hydraulic conductivity. The maintenance of adequate bulk density is an important objective in agriculture. The soil bulk density varied with soil moisture (Figure 8) and decreased by 9 to 22% (after 1st, 2nd and 3rd hydration) at PHNC concentrations 0.1 to 0.4 w/w%, respectively, probably due to the swelling of soil with the incorporated superabsorbent polymer embedded with potassium humate. The decrease in soil bulk density to different extent is in agreement with the studies appraised by Liu *et al*. [19]. The soil porosity increased by 9 to 36% (Figure 9) at hydrogel concentrations 0.1% to 0.4 w/w%.

### 2.5. Effect of PHNC on Hydraulic Conductivity

**Figure 10:**
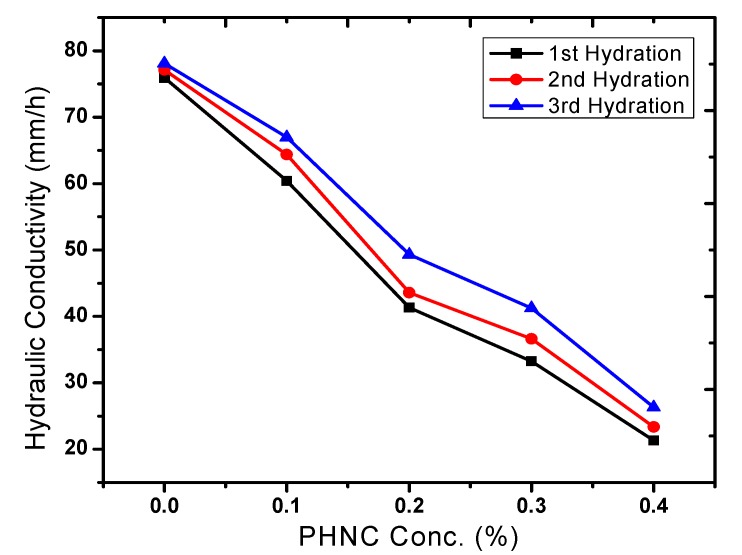
Effect of PHNC on soil hydraulic conductivity.

The addition of 0.1, to 0.4 w/w% of PHNC reduced the hydraulic conductivity 19 to 65% (after 1st, 2nd and 3rd hydration). A remarkable decrease in hydraulic conductivity (Figure 10) was observed with the increase in the concentration of PHNC super absorbent hydrogel nanocomposite. Studies by Bhardwaj *et al*. [25] and El-Shafei *et al*. [26] in sandy soils showed almost similar effects as those observed in the recent study. However, they applied polyacrylamides/gel-conditioner in sandy soils and sprinkler irrigation system. The newly synthesized PHNC enhanced the moisture retention of sandy loam soils and plant available water significantly, thereby slowing down the rate of moisture loss, due to which a delay of 6 to 9 days in wilting point was observed. Such a delay in wilting point reduces the water requirement of plants [2,27,28].

### 2.6. Seed Germination and Seedling Growth of Wheat

The water potential of soil strongly affects seedling emergence and plant establishment [29]. The enhancement in soil moisture retention with superabsorbent hydrogel amendments, improvement in seed germination and seedling growth, have been reported in literature, however there are variations due to the individual superabsorbent hydrogel materials [2]. In the present study seed germination was considerably higher in 0.2 to 0.4% PHNC amended soils as compared to control. The wheat seedling growth was enhanced by the addition of PHNC. Besides, wheat shoot length was significantly higher at 0.3 and 0.4% PHNC compared with other PHNC levels. The addition of 0.3% and 0.4% PHNC in sandy loam soil significantly increased the fresh and dry weights of wheat shoots (Table 1).

**Table 1:** Effect of different levels of PHNC on seed germination and seedling growth (21 days) of wheat (*Triticum aestivum* L.) in sandy loam soils.

Nanocomposite level (%)	Seed germination (%)	Shoot length (cm)	Shoot fresh weight (mg)	Shoot dry weight (mg)
0	80.20 ± 1.61 ^a^	15.24 ± 1.74 ^b^	85.75 ± 2.10 ^c^	16.11 ± 1.82 ^b^
0.1	91.50 ± 1.72 ^ab^	21.76 ± 1.83 ^ab^	92.75 ± 3.45 ^b^	22.45 ± 1.92 ^b^
0.2	92.00 ± 1.75 ^a^	24.61 ± 2.10 ^ab^	112.75 ± 3.26 ^a^	26.50 ± 1.81 ^a^
0.3	96.40 ± 2.10 ^ab^	27.36 ± 2.05 ^a^	131.75 ± 4.25 ^a^	29.20 ± 2.30 ^a^
0.4	98.10 ± 2.25 ^ab^	29.80 ± 2.27 ^a^	162.00 ± 505 ^ab^	35.42 ± 2.61 ^ab^

Mean with same letters within the same column indicate non significant (*P* > 0.05) differences among PHNC concentrations.

### 2.7. Effect of PHNC on Permanent Wilting Point

The maintenance of proper soil moisture is a prerequisite for soil water retention for horticulture purposes. The moisture contents of soil increased with amendment of PHNC at field capacity of sandy loam soils. Hence a significant increase in plant available soil water (AW) was observed with the addition of PHNC compared with untreated soils (Figure 5). At the same time, the amendment with PHNC decreased the hydraulic conductivity and slowed the rate of soil moisture loss thereby delaying the wilting of seedlings. The onset of permanent wilting point (PWP) was delayed by 6 to 9 days with PHNC concentration 0.1 to 0.4%, respectively. Similar effect on the wilting point of barley and chickpea has been reported by Akhtar *et al*. [2] in loam soil.

### 2.8. General Discussion

In the present study the effect of PHNC on different attributes of soil water retention of sandy loam soil amended with different concentrations (0.1, 0.2, 0.3 and 0.4 w/w%) of PHNC was estimated at field capacity (0.03 MPa pressure). The results obtained (Figure 6, Figure 7, Figure 8, Figure 9, Figure 10) reveal that the applied PHNC had a positive effect towards improving the soil characteristics, *i.e*., the values of bulk density and hydraulic conductivity were decreased, whereas total porosity and moisture retention at field capacity were increased with increasing the concentration of PHNC in sandy loam soil. In comparison to our previous study on sandy loam soil amended with SHNC (when moisture was retained for three weeks only) [30], the same soil amended with the newly developed PHNC retained the moisture for more than four weeks. This increase may be attributed to the increase of storage pores in the sandy loam soil which can be regarded as an index of an improved soil structure. The pronounced decrease in hydraulic conductivity of the sandy loam soil may be attributed to the creation of micropores, and the dominance of meso- and micropores. These results are in agreement with those of El-Fayoumy and Ramadan [31] who applied organic soil conditioners surpassed K-humate for improving the soil hydrophysical properties. This was true, since the active -OH and -COOH represent pronounced values and had a profound effect on soil structure as reported by Moustafa *et al.* [32].

Data illustrated in Figure 6, Figure 7, Figure 8, Figure 9, Figure 10 revealed that potassium humate containing basically humic acid when added as individual treatment or combined with other organic soil conditioners surpassed the other treatments for enhancing the availability of essential plant nutrients (N, P, K, Fe, Mn and Zn). This is true, since humic acid partially is capable to retain nutrients for growing plants, where it can act as complexing agent [33]. Enhanced plant growth with the addition of humic substances in soil is related to increase micronutrient availability especially that of iron and zinc. Soil pH and organic matter content significantly affect the solubility of Fe, Mn, Zn and Cu [34].

Humic acid can incorporate iron into the chelate, maintaining its availability to plants, even in insoluble form [35]. Therefore, these chelating agents, through active groups for micronutrients, are considered as a storehouse with easily mobile or available to uptake by plant roots, and in turn reflect positively on development of yield and its attributes for the studied crops.

It is worth mentioning that the positive effect of organic soil conditioners may be due to these organic soil amendments which enhanced crop production and fertilizer uptake by plants through the improvement of hydrophysical properties and thus increased soil ability to supply plants with their requirements of water and air which, consequently, stimulates root growth and the activities of beneficial microorganisms [36].

The above mentioned results indicated that the organic soil amendments affect directly or indirectly the plants nutrients uptake. This means that the applied organic soil amendments are considered as a storehouse with easily mobile or available nutrients to be taken by plant roots. Consequently, these benefits are reflected positively on development of yield. Also, these findings suggest an important role for K-humate in improving the efficiency of nutrient uptake, and in turn increasing the quantity and quality of wheat. The present results confirmed the findings of Mackowiak [33] and Madlain [37] who reported that the beneficial effect of humic acid on dry matter yields may be attributed to improving the bio-availablity of micronutrients by complexion, which prevent early micronutrient deficiency.

## 3. Experimental

### 3.1. Materials

All the chemicals including zinc oxide, aluminum oxide, sodium hydroxide, ferric oxide, acrylic acid, acrylamide, potassium persulphate, potassium metabisulphite and potassium humate were analytical reagent grade purchased from Sigma Chemical Co. (St. Louis, MO, USA). Sandy loam soil (sand 59%, silt 21%, clay 19%, pH 7.5, EC 1.92 dS/m) was collected from the Postgraduate Agricultural Research Station (PARS), Jhang Road, Faisalabad (31°26'N, 73°06'E), Pakistan. The soil was air dried, ground, and passed through a 2 mm sieve. The soil fractions of less than 2 mm were used in the experiments.

### 3.2. Methods

#### 3.2.1. Synthesis of Nano-sized AlZnFe_2_O_4_

AlZnFe_2_O_4_ nano powder was synthesized using the ball milling technique. 5.1 g Al_2_O_3_, 4.05 g ZnO and 16 g Fe_2_O_3_ in the molar ratios of Al_2_O_3_:ZnO:Fe_2_O_3_ (0.5:0.5:1) were dried at 100 °C for 2 h in an electric oven. After cooling to room temperature these were ground to a fine powder in an agate pastel and mortar for 30 min. This fine powder was fed to a ball mill 250 mm long 100 mm wide with glass balls of 15 mm dia. The mass to ball ratio 1:10. The material was ball milled at 100 rpm for 24 h. The ball milled powder was calcined at 600 °C for 4 h. in a muffle furnace. The sample was cooled to room temperature and again ground in the pestle and mortar [38,39,40].

#### 3.2.2. Synthesis of PHNC

The method of Liu and Rempel [41] was followed with some modifications for the preparation of poly(AAm-*co*-AA) AlZnFe_2_O_4_/K-H superabsorbent hydrogel nanocomposite (PHNC). Distilled water (200 mL), acrylic acid (23 g) and acrylamide (2 g) were placed in a flask fitted with a mechanical stirrer, condenser and thermometer. AlZnFe2O4 (1.25 g), potassium humate (1.5 g), Triton X-100 (0.05 g) and diethylene glycol (2.5 g) were also dissolved in the monomer solution by stirring for 30 m. Then potassium persulfate (0.1 g) and potassium metabisulfite (0.04 g) were added and stirred in the flask that was heated to 70 °C. Sodium hydroxide solution was added to the reaction mixture to adjust pH to 4.5. The temperature of the resulting solution was raised to 75 °C and maintained for 2 h. Then the mixture was cooled down to 45 °C and 6.2 mL of 37% formaldehyde was added and stirred for 30 m. Again the reaction mixture was heated to 75 °C for 2 h. The polymer thus formed was precipitated with methyl alcohol, washed with ethyl alcohol, dried at 80 °C and ground.

#### 3.2.3. Physical Characterization

The surface morphology and size of the AlZnFe_2_O_4_ nanoparticles were studied by scanning electron microscope (SEM, JSM-7401F JEOL Ltd., Akishima, Japan) as shown in Figure 1. The chemical composition of the nanoparticles was determined by Energy Dispersive X-ray analysis using Inca-FET-3, Oxford Instruments (UK) Ltd. (Figure 2). The dried SHNC samples were characterized by FTIR spectroscopy using an equipment Tensor-27, Bruker Optics (Ettlingen, Germany) in the scanning range 4000 to 500 cm^−1^.

#### 3.2.4. Measurement of the Water-Retention Properties in Sandy Loam Soil

In order to evaluate water retention properties, sandy loam soils was amended with different concentrations (0.1 to 0.4 w/w%) of PHNC. The amended soils were placed in ventilated paper cups and then100 mL tap water was added initially to the cups (at temperature of 25 °C with relative air humidity equal to 28%) and no water was added after initial watering. The weights of the pots were recorded daily until no noticeable weight loss was observed and water retention ratio was calculated [2,42,43].

#### 3.2.5. Soil pH and EC Measurement

The soil pH and EC, was measured with a Corning-130 pH meter and WTW conductivity meter (model LF-530), respectively. Soil samples were collected from 0–15, 15–30 and 30–45 cm depths (an effective root zone).

#### 3.2.6. Measurement of Bulk Density and Porosity

The soil sample was collected from the depth of 0–15 cm, 15–30 cm, 30–45 cm with core auger (core sampler) and dried in oven for 24 h at 105 °C to determine its oven dry weight (Ws) and calculated soil bulk density using the following relationship:

B.D = Ws/Vt(1)

where B.D = Bulk density of soil (g/cm^3^); Ws = Weight of oven dry soil (g) and Vt = Volume of soil (cm^3^).

#### 3.2.7. Evaluation of Hydraulic Conductivity

The method of Moutier *et al*. [44] with minor changes was followed for the measurement of hydraulic conductivity of soil amended with PHNC and 10 cm long and 5 cm wide Perspex cylinder fitted with a fine metal mesh at the bottom was used to determine the HC. Metal mesh was covered with 5 mm layer of coarse sand. PHNC amended soil (150 g) was put in the cylinder, pressed to a bulk density of 1.5 g/cm^3^ and covered with a filter paper. A peristaltic pump was used to flow tap water (EC = 0.83 dS/m) from the bottom at the rate of 40–50 mm/h. When the water level reached the top of the column the variations in the volume of the soil were calculated by measuring the height of the soil column. The flow direction was reversed and a hydraulic head 0.45 m was applied to leach the tap water from the column. The height of the soil column was monitored during leaching, leachate was collected and hydraulic conductivity was calculated.

#### 3.2.8. Determination of Permanent Wilting Point

The permanent wilting point is the approximate soil water content at which a plant cannot exert enough energy to extract sufficient water from the soil to meet its requirements for survival. Adding water usually does not revive the plant, or if it does, the plant is seriously stunted and probably will not produce an economic yield. Besides, permanent wilting point is the lower limit of plant available soil water which depends upon both plant and soil characteristics and is usually taken as the soil water content at 15 bars of tension [45]. To determine the permanent wilting point of the sandy loam soil, different samples were taken from the top (0–15 cm) layer of the soil after two days of irrigation. These samples were taken from the same points from where, previously samples were taken for determining bulk density. Soil water content at wilting point was determined using pressure membrane apparatus. Soil was filled in the rings and left in a tray of water overnight to be saturated with capillary water. The rings with soil were shifted to the pressure membrane apparatus. Pressure of 1500 kPa was applied until it reached equilibrium (no more water coming out). The soil water contents of the samples were determined using gravimetric method and converted to volumetric basis.

#### 3.2.9. Determination of Total Available Water

The total available soil water (TAW) was calculated with equation:

TAW = FC – WP(2)

where TAW is total available water; FC is field capacity and WP is the wilting point.

#### 3.2.10. Seed Germination and Seedling Growth Assessment in Amended Soil

Wheat (*Triticum aestivum* L.) was selected for the study keeping in view the importance for humanity. Triplicate pots were filled with sandy loam soil amended with 0.1%, 0.2%, 0.3% and 0.4% PHNC. Four seeds of wheat were sown in pots and placed in a growth chamber at 28 ± 2 °C. Triplicate pots of soil without PHNC were kept as control. The percentage of germinated seeds was noted up to two weeks. Shoot emergence was taken as an indicator of seed germination. No water was applied except the initial saturation of the pots and experiment was finished upon the appearance of wilting of seedlings for the first time. After harvesting shoot fresh weight and length were recorded. The dry mass of wheat plant was determined after drying at 70 °C for 24 h.

#### 3.2.11. Statistical Analysis

Data were analyzed using two-way analysis of variance ANOVA using Minitab 2000 Version 13.2 statistical software (Minitab Inc., Centre County, PA, USA) at 5% significance level.

## 4. Conclusions

The results from this study show that the water absorbency of the sandy loam soil is improved by the simultaneous introduction of AlZnFe_2_O_4_ and potassium humate into a poly(AA-*co*-AAM) and novel poly(AA-*co*-AAm)/AlZnFe_2_O_4_/K-H superabsorbent hydrogel nanocomposite (PHNC) had profound effects on physical properties of sandy loam soil. The moisture retention and soil porosity was increased significantly with the application of PHNC and the hydraulic conductivity and soil bulk density decreased relative to the control at different PHNC concentrations. The amount of plant available water significantly increased, hence seed germination and seedling growth of wheat was improved in the amended soil. The PHNC amendment caused a considerable delay in wilting of seedlings growth as compared to control conditions. The soil amendment with PHNC practically ensured improvement of soil moisture retention and seed germination. These findings propose that moisture level, availability of nutrients and soil type have a significant effect on the plant establishment and crop yield. According to these results it can be suggested that usage of nano-superabsorbent (hydrogel) can reduce the harmful effects of drought and improves plant establishment. The current work may be helpful for identifying the best soil agro-management practices of some newly reclaimed soils for maximizing their productivity, especially for soils capable of retaining neither water nor nutrients for growing plants.
